# Pharmacological Depletion of Retinal Mononuclear Phagocytes Is Neuroprotective in a Mouse Model of Mitochondrial Optic Neuropathy

**DOI:** 10.1167/iovs.67.2.6

**Published:** 2026-02-02

**Authors:** Avital L. Okrent Smolar, Rahul Viswanath, Howard M. Bomze, Ying Hao, Sandra S. Stinnett, Sidney M. Gospe

**Affiliations:** 1 Department of Ophthalmology, Duke University School of Medicine, Durham, North Carolina, United States; 2 Department of Biostatistics and Bioinformatics, Duke University School of Medicine, Durham, North Carolina, United States

**Keywords:** Leber hereditary optic neuropathy (LHON), mitochondria, retinal ganglion cell (RGC), hypoxia, pexidartinib, microglia, complex I, Leigh syndrome

## Abstract

**Purpose:**

The *Vglut2-Cre;ndufs4^loxP/loxP^* mouse strain with retinal ganglion cell (RGC)-specific mitochondrial complex I dysfunction develops severe RGC degeneration by postnatal day 90 (P90), with accompanying retinal mononuclear phagocyte (MNP) accumulation. We have reported that continuous exposure to hypoxia partially rescues RGC death in these mice, with minimal effect on MNP abundance. We hypothesized that pharmacological depletion of MNPs with the colony-stimulating factor-1 receptor inhibitor pexidartinib would enhance RGC neuroprotection by hypoxia.

**Methods:**

Iba1^+^ retinal MNP depletion was assessed in C57Bl/6J mice fed control or pexidartinib-infused chow beginning at P25. Subsequently, *Vglut2-Cre;ndufs4^loxP/loxP^* mice and control littermates were raised under normoxia or hypoxia and fed control or pexidartinib chow from P25 to P90. The neuroprotective effect of pexidartinib and hypoxia alone and in combination was assessed by quantifying RGC soma and axon survival in retinal flat mounts and optic nerve cross-sections.

**Results:**

Pexidartinib completely depleted retinal MNPs within 1 week of treatment. Untreated *Vglut2-Cre;ndufs4^loxP/loxP^* mice exhibited the expected approximately 50% reduction of RGC soma and axon survival at P90 (*P* < 0.0001 for both). Hypoxia or pexidartinib monotherapy each reduced RGC degeneration by more than one-half, whereas their combination resulted in complete RGC neuroprotection (*P* < 0.01 for all 3 treatments). Normal myelination patterns were restored in mice receiving dual therapy.

**Conclusions:**

Pexidartinib effectively depletes retinal MNPs and is neuroprotective in the setting of severe RGC mitochondrial dysfunction. This therapeutic effect is additive to that of hypoxia. Combating retinal neuro-inflammation may therefore be a useful adjunct therapy in mitochondrial optic neuropathies like Leber hereditary optic neuropathy (LHON).

Leber hereditary optic neuropathy (LHON) is the most common heritable disease resulting from primary mutations in the mitochondrial DNA (mtDNA).^1^ It is characterized by profound bilateral vision loss in most patients due to rapidly progressive degeneration of retinal ganglion cells (RGCs), which are particularly sensitive to the mitochondrial complex I dysfunction produced by LHON-associated mutations.^2^ Effective treatment for LHON and other mitochondrial optic neuropathies is an important unmet need. Although there is evidence in the literature of some clinical benefit from treatment with oral idebenone to improve mitochondrial function, most patients with LHON continue to have severe visual impairment despite treatment.^3^^–^^5^ Furthermore, although early gene therapy trials for LHON have shown some promise,^6^^,^^7^ a genetically heterogenous disease like LHON would require the development of specific gene therapies for each mutated complex I subunit. The identification of an effective mutation-independent treatment strategy would be a significant advancement for patient care.

In an effort to develop an optimal preclinical mouse model of rapidly progressive mitochondrial optic neuropathy, we have generated a genetically modified mouse line in which severe complex I deficiency is induced within RGCs in a cell-specific manner.^8^ This model utilizes Cre recombinase driven by the Vglut2 promoter to delete *floxed* alleles of the nuclear-encoded complex I accessory subunit *ndufs4* within RGCs. Deletion of this gene, which is mutated in some forms of Leigh syndrome,^9^ destabilizes complex I and decreases its enzymatic activity by >50% in the retina and other tissues.^10^^–^^12^ This severe compromise of complex I function results in rapid degeneration of RGCs that begins at approximately postnatal day 45 (P45) and becomes profound by P90.^8^ Additionally, as an indicator of retinal neuro-inflammation, the mice exhibit inner retinal accumulation of Iba1^+^ mononuclear phagocytes (MNPs), representing proliferation of native microglia and/or infiltration of macrophages derived from peripheral monocytes. The onset of acute RGC loss just after the mice reach sexual maturity is akin to many human cases of LHON and supports the use of this mouse line as a preclinical model for the disease.

We have previously reported that when the *Vglut2-Cre;ndufs4^loxP/loxP^* mice are raised under continuous hypoxia (11% O_2_) beginning at P25, they exhibit marked rescue of RGC axons and somas, with a neuroprotective effect persisting to at least P90.^13^ However, despite reducing RGC degeneration, hypoxia has minimal, if any, effect on the retinal accumulation of MNPs.^13^ Interestingly, it has recently been reported that pharmacological depletion of MNPs results in a significant prolongation of lifespan and inhibition of brain lesion formation in a Leigh syndrome mouse model with global deletion of *ndufs4*.^14^^,^^15^ Furthermore, retinal MNPs have been directly implicated in the pathogenesis of RGC loss in animal models of another optic neuropathy, glaucoma.^16^^,^^17^ Therefore, we were interested to investigate whether depletion of MNPs could reduce RGC degeneration in our mitochondrial optic neuropathy model. To test this, we treated the mice with pexidartinib (PLX3397), an inhibitor of the colony-stimulating factor-1 receptor (CSF-1R), a key mediator of survival signaling for MNPs, both native microglia and infiltrating MNPs derived from circulating monocytes.^18^^,^^19^ Herein, we report that systemic treatment with pexidartinib causes rapid and profound elimination of MNPs from mouse retinas and has a neuroprotective effect on *ndufs4*-deficient RGCs that is equivalent to that of continuous hypoxia. Most notably, the salutary effect of pexidartinib was additive to that of hypoxia, with a complete rescue of RGC somas and axons observed in mice receiving both therapies.

## Methods

### Animals

All animal experiments adhered to the ARVO Statement for the Use of Animals in Ophthalmic and Vision Research, following a protocol approved by the Institutional Animal Care and Use Committee of Duke University. Wild type C57Bl/6J mice were purchased from Charles River Laboratories (Wilmington, MA, USA). *Vglut2-Cre;ndufs4^loxP/loxP^* mice and control littermates were generated as previously described^8^ and maintained on a C57Bl/6J background.

Starting at P25, shortly after weaning, the mice were given ad libitum access to D11112201 OpenStandard diet (Research Diets, Inc., New Brunswick, NJ, USA) with or without 400 mg/kg of pexidartinib (PLX3397; MedKoo Biosciences, Durham, NC, USA). This was the sole food source of the mice until euthanasia for collection of ocular tissues.

### Continuous Hypoxia

Hypoxia treatment was performed as previously described.^13^ Briefly, mouse cages assigned to the hypoxia groups were kept within a hypoxia chamber (A-Chamber animal cage enclosure; BioSpherix, Ltd., Parish, NY, USA) with ambient PO_2_ reduced to 11% by pumping in nitrogen gas to displace the oxygen. The mice were maintained in the hypoxia chamber until P90, under a 12-hour light/dark cycle. Cages with cohorts maintained under normoxia remained in their standard racks in the same animal facility.

### Antibodies

The following antibodies were used for immunofluorescence experiments: rabbit polyclonal anti-RBPMS1 (1:1000, NBP2-20112; Novus Biologicals, Englewood, CO, USA), rabbit polyclonal anti-Iba1 (1:1000, 019–19741; Fujifilm Wako Chemicals Corporation, Richmond, VA, USA), and mouse monoclonal anti-Tuj1 (1:5000; MAB11905; Thermo Fisher Scientific, Waltham, MA, USA). Secondary antibodies against the appropriate species conjugated to Invitrogen Alexa Fluor 488, 568, or 647 (1:5000 dilution) were purchased from Thermo Fisher Scientific. Cell nuclei were stained using Hoechst 33342 (5 µg/mL; Thermo Fisher Scientific).

### Histological Techniques

Immunofluorescence analyses were performed as previously described.^8^ Briefly, enucleated eyes obtained from euthanized mice were fixed overnight in 4% paraformaldehyde. Flat mounts were made from isolated retinas by making 4 radial cuts from the edge to the equator of each retina and then blocked in 5% donkey serum in PBS with 0.3% Triton X-100. The retinas from C57Bl/6J mice were co-incubated with anti-Iba1 and anti-Tuj1 primary antibodies. For the RGC rescue experiment, one quadrant from each retina was removed and incubated with anti-Iba1 and anti-Tuj1 primary antibodies, whereas the remaining three quadrants were incubated in anti-RBPMS1 primary antibody. After 5 days of incubation at 4°C, the retinas were washed and incubated with secondary antibodies in block overnight at 4°C. The retinas were then washed, mounted on glass slides with the RGC layer facing up in Fluoromount-G (SouthernBiotech, Birmingham, AL, USA) and coverslipped.

Images were acquired on a confocal microscope (Eclipse 90i and A1 confocal scanner; Nikon, Tokyo, Japan) with a 60 × objective (1.4 NA Plan Apochromat VC; Nikon) using Nikon NIS-Elements software. Images 45,000 µm^2^ in area were obtained in each quadrant at 3 distances from the optic nerve head: 0.5, 1.0, and 1.5 mm. For RGC soma quantification, images were obtained as z stacks spanning the ganglion cell layer. For MNP quantification, z stacks were obtained from the base of the inner nuclear layer to the inner (vitreous) surface of the retina. RBPMS1^+^ RGC somas and Iba1^+^ MNPs were manually counted using ImageJ software (National Institutes of Health, Bethesda, MD, USA). For each retina, the abundance of each cell type was averaged among all quadrants at each distance from the optic nerve head.

To assess RGC axons, optic nerves were obtained from the same mice and fixed in 2% paraformaldehyde and 2% glutaraldehyde in PBS for 2 hours at room temperature. Samples were embedded in EMbed 812 resin mixture (Electron Microscopy Sciences, Hatfield, PA, USA) and sectioned on an ultramicrotome (LKB Ultratome V; Leica, Wetzlar, Germany) using a glass knife. Cross-sections of 0.27-µm thickness were stained with 1% methylene blue. For each optic nerve specimen, 3 cross-sections were imaged using a Nikon ECLIPSE Ti2 microscope and NIS-Elements imaging software, with sufficient images obtained using a 60× (oil) objective to cover the entire area of the nerve cross-section. The images were stitched into a composite image representing the entire cross-section. Axon count analysis was performed using the AxoNet plugin for ImageJ software.^20^ The final axon count was divided by the total optic nerve area to determine the mean axon density and then averaged over the three cross-sections for each nerve.

For ultrastructural analysis, the same optic nerve specimens were thinly sectioned (60–80 nm), collected on copper grids, counterstained with uranyl acetate and Sato’s lead, and then examined using a transmission electron microscope (JEM-1400; JEOL USA, Peabody, MA, USA) at 60 kilovolts (kV). Images were collected using an Orius charge-coupled device camera (Gatan, Pleasanton, CA, USA).

Select fixed optic nerves were cryoprotected in 30% sucrose and embedded in optimal cutting temperature (OCT) medium (Tissue-Tek, Sakura Finetek). Transverse cross-sections and longitudinal sections of 15-µm thickness were collected using a cryostat microtome (Microm HM 550; Thermo Fisher Scientific). Sections were rehydrated with PBS and stained with anti-Iba1 antibody and DAPI, as above, treated with the primary antibody overnight at 4°C. The sections were imaged by confocal microscopy as above.

### Experimental Design and Statistical Analysis

Quantitative assessment of MNP depletion from the retinas of male and female wild type C57Bl/6J mice was performed on both retinas of 3 to 4 mice from each group at each time point. Quantification of RGC soma and axon survival and of MNP abundance was performed on *Vglut2-Cre;ndufs4^loxP/loxP^* mice and *Vglut2-Cre;ndufs4^loxP/+^* littermate controls, with both sexes represented in each group. In these experiments, 10 to 16 retinas or optic nerves were analyzed for each combination of genotype, O_2_ concentration, and chow. In order to avoid individual housing of mice and to promote equal representation of sexes in each experimental group (aiming for at least 40% representation of each sex), the assignment of individual animals to each group was made after genotyping, without randomization. Even after unexpected attrition of some animals, each sex comprised at least one-third of every group. While performing RGC soma and axon counts, the observer was masked to the identity of each sample.

Statistical analyses were performed using SAS version 9.4 (SAS Institute, Cary, NC, USA). When comparing the *Vglut2-Cre;ndufs4^loxP/loxP^* mice with homozygous deletion of *ndufs4*, a 2-way analysis of variance (ANOVA) was used to assess the effects of O_2_ concentration, diet, and their interaction on the outcome variables (soma counts, axon counts, and MNP counts) while adjusting for sex. Because both eyes of each mouse were included, generalized estimating equations (GEEs) with an identity link and exchangeable correlation structure was used to adjust for the correlation between eyes. For pair-wise comparisons, the Z-test of difference in means between groups was used. Bonferroni adjustment was made for each analysis with six pair-wise tests. The adjusted alpha-level for significance was *P* < 0.008. For soma counts, separate analyses were conducted for each distance from the optic nerve head (0.5, 1.0, and 1.5 millimeters).

Because the control *Vglut2-Cre;ndufs4^loxP/+^* heterozygous littermates received either no treatment or both treatments (hypoxia and pexidartinib), they could not be included in the 2-way ANOVA. However, additional analyses were carried out to compare specific subgroups, in order to determine the extent of RGC degeneration in untreated knockout mice compared with untreated heterozygotes, the completeness of RGC rescue in knockout mice receiving both treatments, and the accumulation of MNPs in knockout retinas compared with controls. These pair-wise comparisons were made using GEEs with an identity link and exchangeable correlation structure. No adjustment of alpha-level was made for these subgroup comparisons. In all figures, data are presented graphically as mean ± standard error the mean (SEM), and individual data points on the bar graphs depict data from each retina or optic nerve analyzed.

## Results

**Figure 1.**
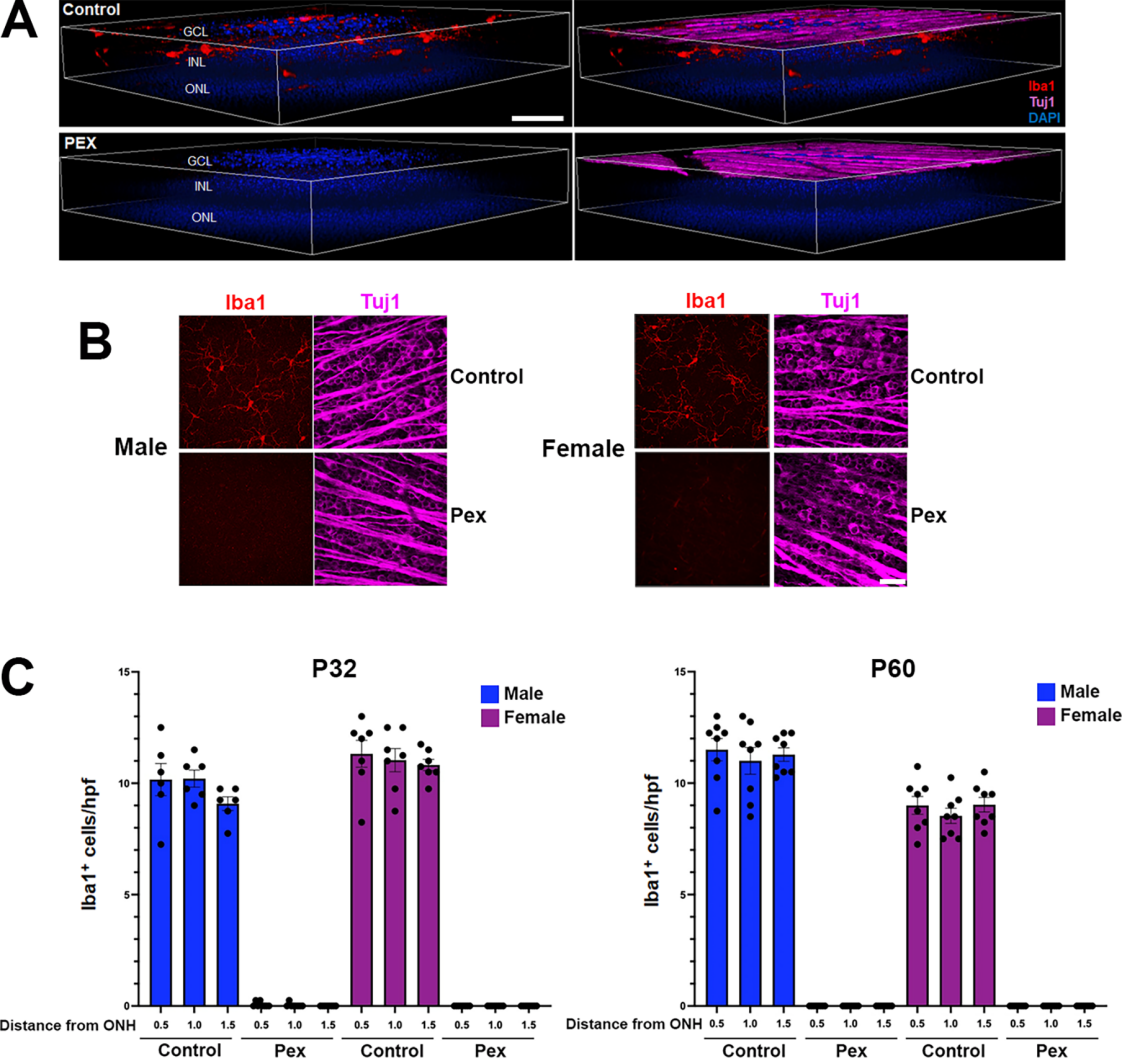
**Systemic administration of pexidartinib efficiently depletes retinal mononuclear phagocytes from wild type mice.** (**A**) Representative 3-dimensional reconstructions of confocal microscopy z-stacks of retinal flat mounts from wild type C57Bl/6J mice administered control (*top*) or pexidartinib-infused chow (*bottom*) for 1 week. Mononuclear phagocytes (MNPs) are immunolabeled in *red* for Iba1, and the nuclear layers of the retina are labeled with DAPI (*blue*). The *panels to the right* additionally show immunolabeling for β3-tubulin (Tuj1; *magenta*) to highlight the retinal nerve fiber layer comprised by RGC axons. Pexidartinib treatment eliminated Iba1^+^ MNPs from both the inner retina (those residing between the inner nuclear layer and ganglion cell layer) and the outer retina (those deep to the inner nuclear layer). GCL, ganglion cell layer; INL, inner nuclear layer; ONL, outer nuclear layer. *Bar* = 40 µm. (**B**) Representative flat mounts of P32 retinas of wild type male and female mice, after 1 week of treatment with control or pexidartinib chow. MNPs are labeled with Iba1 (*red*) and the nerve fiber layer with Tuj1 (magenta). *Bar* = 40 µm. (**C**) Quantification of the abundance of Iba1^+^ MNPs per high power field (hpf) in the inner retina at distances of 0.5, 1.0, and 1.5 mm from the optic nerve head, demonstrating near-complete depletion in mice of both sexes treated with pexidartinib. The *left graph* compares mice treated from P25 to P32. The *right graph* compares mice treated for an additional month with control or pexidartinib chow through P60. There were three to four mice per group, with data from each retina shown as individual points. Error bars depict mean ± standard error of the mean (SEM).

Based on two recent reports that treatment of the *ndufs4^−^^/^^−^* mouse model of Leigh syndrome with pexidartinib resulted in a several-fold prolongation of the lifespan of the animals,^14^^,^^15^ we were interested to determine whether the drug would prove neuroprotective in our mouse strain with RGC-specific deletion of the same gene. Pexidartinib is a tyrosine kinase inhibitor that targets CSF-1R to deprive MNPs of a key survival signal. It has been shown to cross the blood-brain barrier, effectively depleting MNPs from the brain as well as retinal tissue.^21^^,^^22^ However, there have been conflicting data regarding whether the efficacy of pexidartinib in depleting Iba1^+^ MNPs from the mouse brain is sex-dependent, with some reports describing a much more pronounced effect in male mice compared with female mice.^23^^,^^24^ In order to determine how rapidly and completely pexidartinib eliminates MNPs from the retina, and whether this would be impacted by the sex of the mice, we first administered the drug to young wild type C57Bl/6J mice beginning just after weaning at P25 via drug-infused chow (400 mg/kg).^24^ Retinal tissue was harvested after either 1 week (P32) or 1 additional month (P60) to determine the extent and durability of MNP depletion. We observed near-complete loss of Iba1^+^ cells from both the inner and outer retina after just 1 week of treatment (Fig. 1A). Because only those MNPs in the vicinity of RGCs would be likely to impact their survival in a mitochondrial optic neuropathy, we limited our quantitative analysis of MNP abundance to those localizing to the inner retina (from the junction of the inner nuclear layer and inner plexiform layer to the internal limiting membrane). With rare exception, we observed complete loss of inner retinal Iba1^+^ cells at P32 in both male and female mice treated with pexidartinib (Figs. 1B, 1C). Continued administration of pexidartinib achieved a durable elimination of MNPs, with none observed in any of the retinas at P60, irrespective of sex (see Fig. 1C).

**Figure 2.**
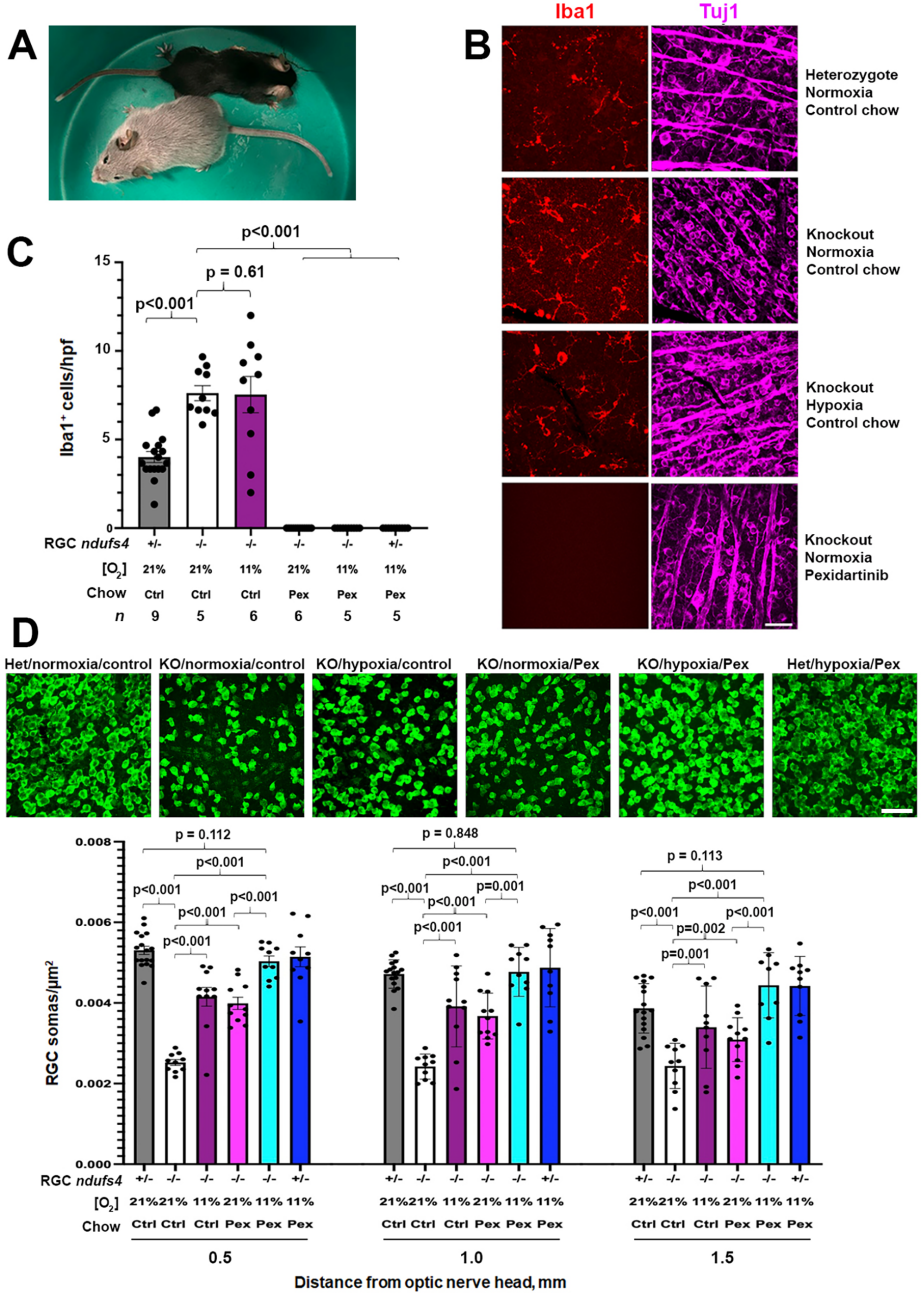
**Pexidartinib treatment of *Vglut2-Cre;ndufs4^loxP/loxP^* mice produces a neuroprotective effect on retinal ganglion cell soma degeneration that is additive to that of hypoxia.** (**A**) Photograph of *Vglut2-Cre;ndufs4^loxP/loxP^* littermates at P90. The *top* mouse received control chow and has become weak, with splayed limbs. The *bottom* mouse received pexidartinib chow and demonstrates a normal posture. Graying of the coat is a side effect of high dose pexidartinib. (**B**) Representative images of Iba1-labeled (*red*) retinal flat mounts from a heterozygous *Vglut2-Cre;ndufs4^loxP/+^* control mouse and from *Vglut2-Cre;ndufs4^loxP/loxP^* mice that are untreated or received hypoxia or pexidartinib treatment. Tuj1 (*magenta*) was used to confirm image capture at the level of RGCs. *Bar* = 40 µm. (**C**) Comparison of Iba1^+^ MNP abundance between experimental cohorts. The *ndufs4^+/−^* indicates deletion of one allele of *ndufs4* from RGCs (*Vglut2-Cre;ndufs4^loxP/+^* mice), and *ndufs4^−^^/^^−^* indicates deletion of both alleles (*Vglut2-Cre;ndufs4^loxP/loxP^* mice). There is an accumulation of Iba1^+^ MNPs in the inner retina in untreated mice with *ndufs4*-deficient RGCs that is unaffected by hypoxia treatment. Complete depletion of MNPs from the inner retina was observed in all cohorts receiving pexidartinib. The number of mice, *n*, analyzed per cohort is reported beneath each bar. Graphs depict mean ± standard error of the mean (SEM), and data from each retina are shown as individual points. The *P* values are shown for post hoc pairwise comparison (Z-test of difference of means) subsequent to ANOVA. (**D**) Representative images of RBPMS1-labeled RGC somas (*green*) obtained at a distance of 1.0 mm from the optic nerve head in retinal flat mounts from each experimental cohort at P90. The graph below demonstrates a significant reduction of RGC soma loss in *Vglut2-Cre;ndufs4^loxP/loxP^* mice treated with hypoxia or pexidartinib; the combined treatment restores RGC survival to the level observed in heterozygous *Vglut2-Cre;ndufs4^loxP/loxP^* control mice that were untreated or received the dual therapy. The number of mice per cohort is the same as in **C**. Graph depicts mean ± SEM, and *P* values reflect post hoc pairwise comparisons of means following ANOVA.

Because RGC loss in the *Vglut2-Cre;ndufs4^loxP/loxP^* mouse model of mitochondrial optic neuropathy does not commence until after P30 and progresses similarly in mice of both sexes,^8^ we proceeded to test the effect of pexidartinib treatment beginning at P25, using both male and female littermates. The mutant mice live longer than global *ndufs4* knockout mice, but do not survive much beyond P90 due to progressive neurological dysfunction arising from complex I deficiency in those glutamatergic neurons in the brain expressing Vglut2-driven Cre recombinase.^8^^,^^25^ We therefore selected P90 as the terminal time point at which ocular tissues would be harvested. Pexidartinib-infused chow or control chow were administered as the sole sources of food to the experimental *Vglut2-Cre;ndufs4^loxP/loxP^* mice and to control *Vglut2-Cre;ndufs4^loxP/+^* littermates that are heterozygous for loss of *ndufs4* and previously shown to be aphenotypic.^13^ In order to compare any neuroprotective effect of pexidartinib to that of hypoxia and to determine any added benefit when the 2 treatments were combined, half of the mice from each group were raised under normoxia (21% O_2_) or continuous hypoxia (11% O_2_) beginning at P25. Whereas the *Vglut2-Cre;ndufs4^loxP/loxP^* mice fed a control diet and raised under normoxia demonstrated the expected severe stiffened posture and ataxia by P90, those treated with pexidartinib and/or hypoxia exhibited a body posture and activity level grossly indistinguishable from their heterozygous littermates, consistent with the neurological improvement reported in studies of global *ndufs4^−^^/^^−^* mice administered either treatment.^15^^,^^26^ All mice treated with pexidartinib exhibited an acquired depigmentation of their hair, previously observed in mice and a known side effect of the drug in humans^15^^,^^27^ (Fig. 2A).

The abundance of Iba1^+^ MNPs in the inner retina was assessed in retinal flat mounts from each group (Fig. 2B). Similar to our prior observations from retinal cross sections, MNPs were observed at a frequency 2-fold higher in untreated *Vglut2-Cre;ndufs4^loxP/loxP^* mice at P90 compared with heterozygous controls, and this increase was not mitigated by hypoxia treatment (Fig. 2C). Pexidartinib treatment resulted in a complete elimination of inner retinal MNPs at P90 in all groups receiving the drug.

Retinal flat mounts were immunolabeled with the RGC marker RBPMS1 to allow quantification of RGC soma survival in each group (Fig. 2D). Consistent with our prior observations, the untreated *Vglut2-Cre;ndufs4^loxP/loxP^* mice exhibited approximately 50% loss of RGC somas at P90 compared with heterozygous littermates. Both continuous hypoxia and pexidartinib significantly reduced RGC soma loss in *Vglut2-Cre;ndufs4^loxP/loxP^* mice at three different distances from the optic nerve head; their effects were additive, as no interaction between the variables was found on 2-way ANOVA (Supplementary Table S1). RGC soma counts were not affected by the sex of the mice. Consistent with our prior observations, continuous hypoxia prevented more than half of RGC attrition in P90 *Vglut2-Cre;ndufs4^loxP/loxP^* mice; pexidartinib treatment was found to promote a similarly robust but incomplete rescue of RGCs (see Fig. 2D; Supplementary Table S2). Most remarkably, the additive neuroprotective effect of dual treatment with hypoxia and pexidartinib fully restored normal RGC soma counts, with mean soma densities 95%, 101%, and 116% of those obtained from heterozygous controls at retinal locations proximal, intermediate, and distal from the optic nerve head, respectively (see Fig. 2D; Supplementary Table S3). Notably, heterozygous mice administered the dual treatment also exhibited normal RGC survival, indicating that neither treatment was deleterious to healthy RGCs without mitochondrial dysfunction.

**Figure 3.**
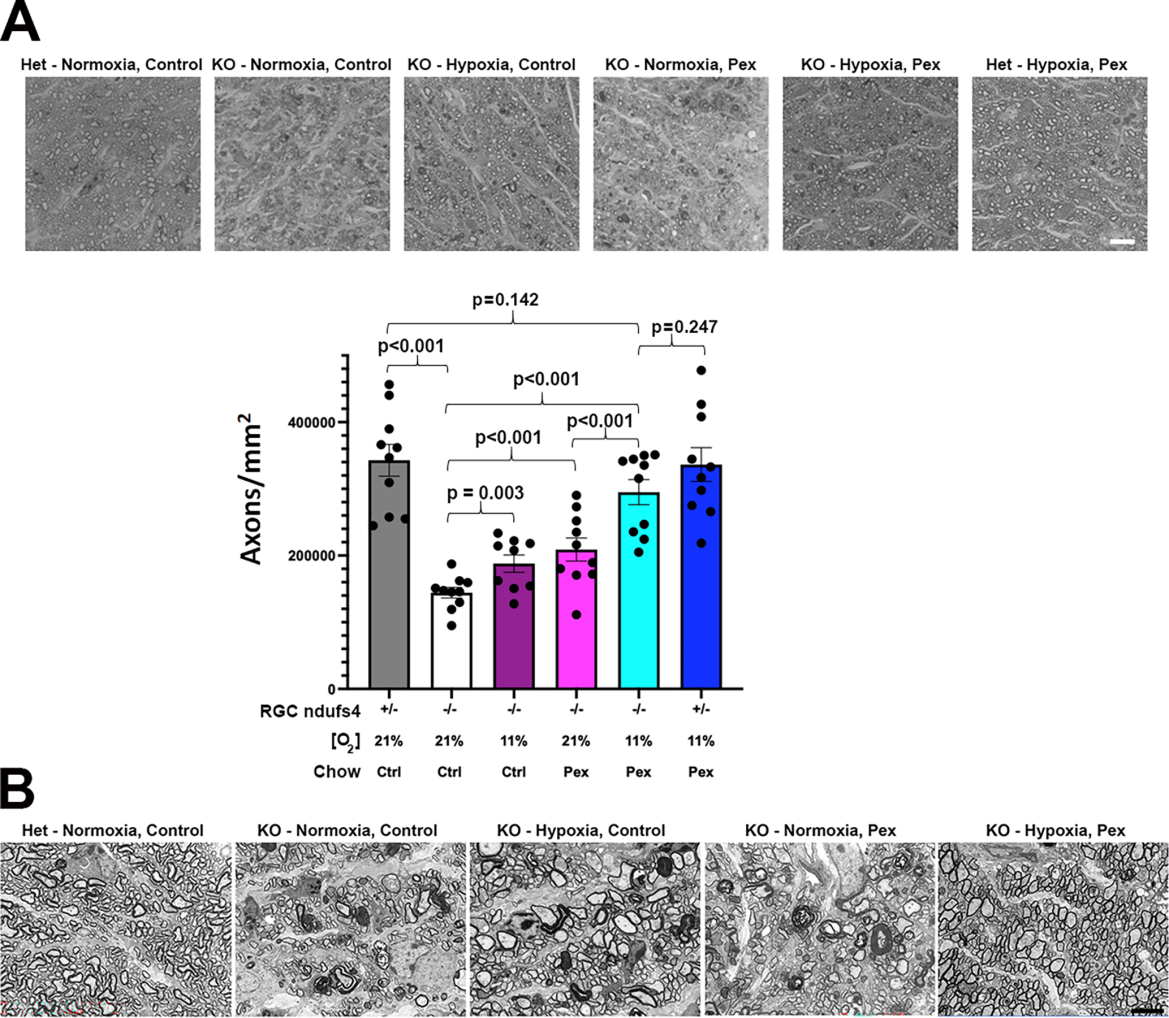
**Hypoxia and pexidartinib reduce RGC axonal degeneration in *Vglut2-Cre;ndufs4^loxP/loxP^* mice in an additive manner.** (**A**) Representative light micrographs of optic nerve cross sections stained with methylene blue. The genotype within RGCs (heterozygous [Het] or knockout [KO] for *nduf4*) and treatment of each cohort is indicated above each panel. *Bar* = 10 µm. The graph below shows RGC axon densities in optic nerve cross sections. The *ndufs4^+/−^* indicates deletion of one allele of *ndufs4* (*Vglut2-Cre;ndufs4^loxP/+^* mice), and *ndufs4^−^^/^^−^* indicates deletion of both alleles within RGCs (*Vglut2-Cre;ndufs4^loxP/loxP^* mice). The O_2_ concentration is depicted for each group, as is the type of chow administered. There were five mice per cohort with data from each optic nerve shown as individual points. Graphs are presented as mean ± standard error of the mean (SEM). The *P* values for post hoc pairwise comparisons following ANOVA are shown above the bars. (**B**) Electron micrographs (5000×) of optic nerve cross sections at P90 demonstrate preservation of normal axon morphology and abundance in *Vglut2-Cre;ndufs4^loxP/loxP^* mice treated with both hypoxia and pexidartinib, whereas those receiving monotherapy demonstrate a partial reduction of abnormal myelination compared with the untreated *Vglut2-Cre;ndufs4^loxP/loxP^* mice. *Bar* = 5 µm.

We then assessed the optic nerves of these mice to assess the neuroprotective effect of each treatment on RGC axons (Fig. 3A). Similar to RGC somas, there was a 58% reduction in axon density in untreated *Vglut2-Cre;ndufs4^loxP/loxP^* mice at P90. RGC axon degeneration was significantly reduced by either continuous hypoxia or pexidartinib (Supplementary Tables S4, S5). Dual therapy again demonstrated additivity, with axon survival restored to levels not significantly different from heterozygous *Vglut2-Cre;ndufs4^loxP/+^* controls receiving either no therapy or the dual therapy (>85% of each; Supplementary Table S6). Optic nerve ultrastructure was then assessed by transmission electron microscopy (Fig. 3B). Characteristic myelination abnormalities were observed in specimens from the untreated *Vglut2-Cre;ndufs4^loxP/loxP^* mice, including thickening or duplication of myelin sheaths or incomplete enclosure of axons by myelin. Dual therapy with hypoxia and pexidartinib prevented the development of pathological myelination patterns; partial improvement was observed when the experimental mice were administered either treatment in isolation.

## Discussion

In this study, we observed that mice undergoing systemic treatment with pexidartinib exhibited a rapid and virtually complete depletion of retinal MNPs within 1 week of starting the therapy. This depletion persisted over 2 months of chronic treatment and was not dependent on the sex of the mice. The drug itself and the consequent loss of retinal MNPs did not adversely affect the survival of RGCs. When applied to mice with RGC-specific complex I dysfunction, pexidartinib produced a significant, although partial, rescue of RGC soma and axon degeneration, similar in magnitude to that observed when the mice were instead treated with continuous hypoxia (11% O_2_). Most remarkably, the neuroprotective effects of each of these treatments proved to be additive, with the combined therapy resulting in essentially complete rescue of RGCs at P90, suggesting that the treatments work through independent mechanisms and that mitochondrial optic neuropathies may respond best to a combinatorial therapeutic approach.

The most intuitive strategy to treating a mitochondrial disease is to mitigate the effects of mitochondrial dysfunction directly. This has been the rationale behind LHON treatments such as idebenone and gene therapy.^28^ Idebenone, a synthetic coenzyme Q10 analog, is thought to promote RGC neuroprotection by delivering electrons to complex III, thereby bypassing the dysfunctional complex I to augment mitochondrial ATP production, while also serving as a potent antioxidant to reduce oxidative stress.^29^ It is commonly administered to patients with LHON within the first several years of vision loss, and studies have suggested a modest benefit in visual outcome when compared to placebo or natural history, possibly dependent on specific mtDNA mutations.^4^^,^^5^ Gene therapy trials have aimed to restore normal complex I function in RGC mitochondria by using adeno-associated virus to deliver wild type versions of mutated mitochondrial genes, thus far limited to two genes, *mtND4* and *mtND1*.^7^^,^^30^ Similar to idebenone, the gene therapies have shown generally positive outcomes, although most subjects have remained legally blind.^6^ Hypoxia, which we have previously described as protective against degeneration of RGC somas and axons in our mouse model of RGC *ndufs4* deficiency,^13^ is also likely to have a direct effect on RGCs. While the neuroprotective mechanism of hypoxia remains under investigation, work by our laboratory and others has suggested that it is independent of activation of the hypoxia-inducible factor pathway.^31^^,^^32^ Rather, neuroprotection by hypoxia likely occurs via reducing the production of reactive oxygen species and/or promoting the forward transfer of electrons within affected mitochondria.^31^^,^^33^ Nevertheless, despite its effectiveness at slowing RGC degeneration, hypoxia has shown no more than minimal impact in reducing accumulation of Iba1^+^ MNPs, at least in the retina.^13^

In contrast to environmental hypoxia, the neuroprotective effect of pexidartinib is likely to be indirect, via its actions on MNPs. In addition to our own observations of retinal MNP accumulation in the RGC-specific *ndufs4* deletion model, prior analysis of the retinas of mice with global deletion of *ndufs4* revealed a substantial increase in the expression of cytokines and other genes related to innate immunity, even prior to the onset of RGC loss.^34^ It should be noted that, in our RGC-specific disease model, the MNPs themselves remain metabolically intact, so their accumulation in the retina is presumably secondary to signaling from stressed RGCs. The enhanced neuroprotection of *ndufs4*-deficient RGCs observed when the mice were administered both hypoxia and pexidartinib most likely reflects the combined benefit of reducing intrinsic metabolic stress within RGCs via hypoxia and eliminating extrinsic cytotoxic effects of accumulating MNPs. Accordingly, a general strategy of mitigating neuroinflammation as an adjunct to boosting mitochondrial function may prove effective in a variety of mitochondrial diseases. In the case of LHON, where continuous hypoxia is impractical for human patients, combining the systemic or intraocular delivery of a CSF-1R inhibitor like pexidartinib with RGC-targeted treatments like gene therapy or idebenone might hold particular promise.

The specific role that MNPs, particularly microglia, play in the pathophysiology of neurodegenerative diseases is controversial. Both adaptive and pathological functions of MNPs have been described in animal models of traumatic brain injury, demyelinating disease, cerebral ischemia, and neurodegenerative pathologies, such as Alzheimer’s disease and Parkinson’s disease.^23^^,^^35^^–^^39^ Similarly, in the retina, MNPs that infiltrate into the subretinal space appear to have a protective role in a mouse model of retinitis pigmentosa by clearing debris and secreting pro-survival signals, whereas in other diseases, like age-related macular degeneration, MNPs in this location may exacerbate photoreceptor degeneration.^40^^,^^41^ The multifaceted roles of MNPs likely influence their contributions to disease processes affecting RGCs as well. Whereas an analysis of two mouse models of glaucoma demonstrated that MNPs adopt neurodegenerative molecular signatures and promote damage to RGCs,^17^ the depletion of MNPs from the retina and optic nerve via CSF-1R inhibition has been reported to exacerbate glaucomatous optic nerve damage in the same two models.^42^^,^^43^ Pharmacological MNP depletion has also been applied to optic nerve crush models, with discrepant reports of improved RGC survival^44^ versus no neuroprotective effect being observed.^45^^,^^46^ Interestingly, when applied to a zebrafish optic nerve crush model, CSF-1R inhibition was found to be detrimental to RGC axonal regeneration following injury.^45^^,^^47^ Despite this ambiguity, our observation that chronic depletion of MNPs improved survival of RGCs with mitochondrial impairment would suggest that, on balance, MNPs exacerbate the pathogenesis of mitochondrial optic neuropathy.

As the previous optic neuropathy studies may have been impacted by variability in the completeness of MNP depletion from the retina and optic nerve following oral administration of CSF-1R inhibitors, it would be worthwhile to determine whether MNP depletion in our model is as robust in the optic nerve as it is in the retina. An examination of a small number of optic nerves not used for plastic sections suggested that by P90 MNPs were strongly depleted by pexidartinib (Supplementary Fig. S1); however, it would be illuminating to perform a more formal quantitative analysis including earlier time points in order to determine the rate and completeness of MNP elimination from the optic nerve. It would also be interesting to investigate the rate at which MNPs repopulate the retina and optic nerve following cessation of pexidartinib and to determine whether intermittent dosing of the drug following initial induction could be similarly neuroprotective in our model.

It should be noted that as an inhibitor of the CSF-1R, pexidartinib deprives immune cells of a critical survival signal, resulting in depletion not only of native microglia but also circulating myeloid cells and a subset of lymphoid cells by suppressing progenitor cell lines in the spleen, bone marrow, and blood.^48^ It is therefore uncertain whether the therapeutic effect of pexidartinib on RGCs in the *ndufs4* mouse model is mediated by depletion of microglia or a prevention of infiltration of circulating immune cells. Defining the relevant MNP population that accumulates in the retinas of mice with RGC-specific complex I deficiency will be an important future direction that could ultimately lead to more targeted therapies. Because overlapping immunophenotypes (including Iba1 positivity) make it challenging to distinguish microglia from infiltrating monocyte-derived MNPs (macrophages) using conventional immunohistochemistry methods, lineage tracing experiments with genetically modified mouse lines (e.g., *CCR2^RFP^* mice with intrinsic labeling of circulating monocytes) may be required for a definitive determination.^49^^,^^50^ Potentially instructive is a recent report that the *Csf1r*ΔFIRE mutation that eliminates microglia but not monocyte-derived MNPs did not reduce the formation of brain lesions and neurologic dysfunction in the Leigh syndrome mouse model with global *ndufs4* deletion.^38^ This may suggest that microglia are of less importance than infiltrating macrophages in driving the pathogenesis of neurodegeneration in mitochondrial diseases.

Finally, it is important to consider that pexidartinib and other drugs targeting CSF-1R do so by functioning as tyrosine kinase inhibitors, raising the possibility of off-target effects. Tyrosine kinases are involved in multiple cellular signaling pathways, including those related to the cell cycle, metabolism, and cell proliferation, differentiation, and survival.^51^ In addition to potently inhibiting CSF-1R, pexidartinib is also known to inhibit the tyrosine kinases c-Kit (which likely accounts for its effect on hair color) and FLT3.^52^^,^^53^ It is clear that future experiments using alternative genetic and pharmacological methods to achieve specific ablation of the various subpopulations of MNPs will be important to determine whether the salutary impact of pexidartinib in the *ndufs4* knockout model is truly a direct consequence of MNP depletion.

An important limitation of our study is that our characterization of RGC survival relied solely on histological analyses of RGC somas and axons. It is conceivable that the increased persistence of *ndufs4*-deficient RGCs in mice treated with pexidartinib could be explained by the loss of MNPs required to clear dead and dying cells rather than by true neuroprotection. Although this might explain some element of the increased RGC counts at P90, we would expect decreased clearance to be of relatively minor importance for two reasons. First, despite pexidartinib by itself producing only a partial reduction of RGC soma and axon loss in the knockout mice, pexidartinib dramatically added to the effect of hypoxia, completely preventing the loss of RGCs that die with hypoxia monotherapy. Second, the marked improvement in the morphology of RGC axon myelination by oligodendrocytes when the mutant mice were treated by pexidartinib would unlikely be explained simply by decreased clearance of dead RGCs. Nevertheless, demonstration of improved function of rescued *ndufs4*-deficient RGCs using electrophysiological methods, such as pattern electroretinography or visual evoked potentials, will be a critical next step to confirm the neuroprotective potential of CSF-1R inhibition.

## Conclusions

The combination of pexidartinib and hypoxia resulted in an additive, complete anatomic rescue of RGCs with complex I dysfunction. Although the precise cellular mechanisms by which each treatment was neuroprotective in this model remain to be determined, the additive effect of the treatments demonstrates that there are at least two independent mechanisms leading to RGC survival. Our observations raise the intriguing prospect that a multimodal approach may be key to optimal management of mitochondrial optic neuropathies like LHON. Although reversing the mitochondrial impairment in RGCs that drives the optic neuropathy is a worthy therapeutic goal, modulation of neuroinflammation may be of similar importance in preventing optic atrophy.

## Supplementary Material

Supplement 1
