# Supplementary material for: Pharmacological Depletion of Retinal Mononuclear Phagocytes Is Neuroprotective in a Mouse Model of Mitochondrial Optic Neuropathy
Source: Invest Ophthalmol Vis Sci. 2026 Feb 2;67(2):6. doi: 10.1167/iovs.67.2.6 (PMC12875350; doi:10.1167/iovs.67.2.6)
Supplement: Supplement 1 [file iovs-67-2-6_s001.pdf]

Supplementary Table S1. P-values of overall tests for RGC soma densities of *Vglut2-Cre;ndufs4*$^{loxP/loxP}$ mice treated with varying O_2 concentrations and chow with and without Pexidartinib (Two-Way ANOVA)

Variable	Distance from Optic Nerve (mm)		
	**0.5**	**1.0**	**1.5**
Oxygen	**<0.001**	**0.002**	**0.002**
Chow	**<0.001**	**0.001**	**0.007**
Oxygen x Chow	0.143	0.496	0.409
Sex	0.997	0.832	0.235

Supplementary Table S2. P-values for pair-wise comparisons of RGC soma densities (differences) of *Vglut2-Cre;ndufs4*$^{loxP/loxP}$ mice receiving different Oxygen-Chow combinations

Comparisons				Distance from Optic Nerve (mm)					
Group 1		Group 2		0.5		1.0		1.5	
**Oxygen**	**Chow**	**Oxygen**	**Chow**	**P-value**	**95% CI***	**P-value**	**95% CI***	**P-value**	**95% CI***
Normoxia	Control	Hypoxia	Control	**<0.001**	-2.08, -1.15	**<0.001**	-2.12, -0.744	**0.001**	-1.62, -0.419
Normoxia	Control	Normoxia	PEX	**<0.001**	-1.78, -1.15	**<0.001**	-1.70, -0.771	**0.002**	-0.985, -0.220
Normoxia	Control	Hypoxia	PEX	**<0.001**	-2.84, -2.18	**<0.001**	-2.87, -1.81	**<0.001**	-2.71, -1.35
Hypoxia	Control	Normoxia	PEX	0.565	-0.362, 0.662	0.632	-0.603, 0.994	0.220	-0.250, 1.09
Hypoxia	Control	Hypoxia	PEX	**0.001**	-1.42, -0.371	0.021	-1.68, -0.140	0.020	-1.87, -0.157
Normoxia	PEX	Hypoxia	PEX	**<0.001**	-1.46, -0.630	**0.001**	-1.77, -0.434	**<0.001**	-2.19, -0.664

* 95% Confidence Intervals constructed for differences in RGC soma densities between the two indicated groups, expressed in units of RGCs x 10^{-3} per mm^2. After applying a Bonferroni correction, a p-value ≤0.008 was required for statistical significance.

Supplementary Table S3. P-values for pair-wise comparisons of RGC soma densities (differences) between *Vglut2-Cre;ndufs4*$^{loxP/loxP}$ mice and *Vglut2-Cre;ndufs4*$^{loxP/+}$ controls receiving different Oxygen-Chow combinations

Subgroups	Distance from Optic Nerve (mm)					
	0.5		1.0		1.5	
	**P-value**	**95% CI***	**P-value**	**95% CI***	**P-value**	**95% CI***
Het/Normoxia/Control Chow vs KO/Normoxia/Control Chow	**<0.001**	2.52, 3.08	**<0.001**	2.04, 2.55	**<0.001**	1.05, 1.81
Het/Normoxia/Control Chow vs KO/Hypoxia/PEX	0.112	-0.067, 0.644	0.848	-0.529, 0.435	0.113	-1.22, 0.129

* 95% Confidence Intervals constructed for differences in RGC soma densities between the two indicated groups, expressed in units of RGCs x 10^{-3} per mm^2.

Supplementary Table S4. P-values of overall tests for RGC axon densities of *Vglut2-Cre;ndufs4*$^{loxP/loxP}$ mice treated with varying O_2 concentrations and chow with and without Pexidartinib (Two-Way ANOVA)

Variable	P-value
Oxygen	**0.002**
Chow	**0.001**
Oxygen x Chow	0.227
Sex	0.277

Supplementary Table S5. P-values for pair-wise comparisons of RGC axon densities (differences) of *Vglut2-Cre;ndufs4*$^{loxP/loxP}$ mice receiving different Oxygen-Chow combinations

Group 1		Group 2			
**Oxygen**	**Chow**	**Oxygen**	**Chow**	**P-value**	**95% CI***
Normoxia	Control	Hypoxia	Control	**0.003**	-90956, -17990
Normoxia	Control	Normoxia	PEX	**<0.001**	-91552, -31975
Normoxia	Control	Hypoxia	PEX	**<0.001**	-190648, -110903
Hypoxia	Control	Normoxia	PEX	0.710	-45734, 31154
Hypoxia	Control	Hypoxia	PEX	**<0.001**	-144127, -48477
Normoxia	PEX	Hypoxia	PEX	**<0.001**	-128932, -49092

* 95% Confidence Intervals constructed for differences in RGC axon densities between the two indicated groups, expressed in units of axons per mm^2. After applying a Bonferroni correction, a p-value ≤0.008 was required for statistical significance

Supplementary Table S6. P-values for pair-wise comparisons of RGC axon densities (differences) between *Vglut2-Cre;ndufs4*$^{loxP/loxP}$ mice and *Vglut2-Cre;ndufs4*$^{loxP/+}$ controls receiving different Oxygen-Chow combinations

Subgroups	P-value	95% CI*
Het/Normoxia/Control Chow vs KO/Normoxia/Control Chow	**<0.001**	142744, 255515
Het/Normoxia/Control Chow vs KO/Hypoxia/PEX	0.142	-16176, 112621
Het/ Hypoxia/PEX vs KO/Hypoxia/PEX	0.247	-28651, 111396

* 95% Confidence Intervals constructed for differences in RGC axon densities between the two indicated groups, expressed in units of axons per mm^2.

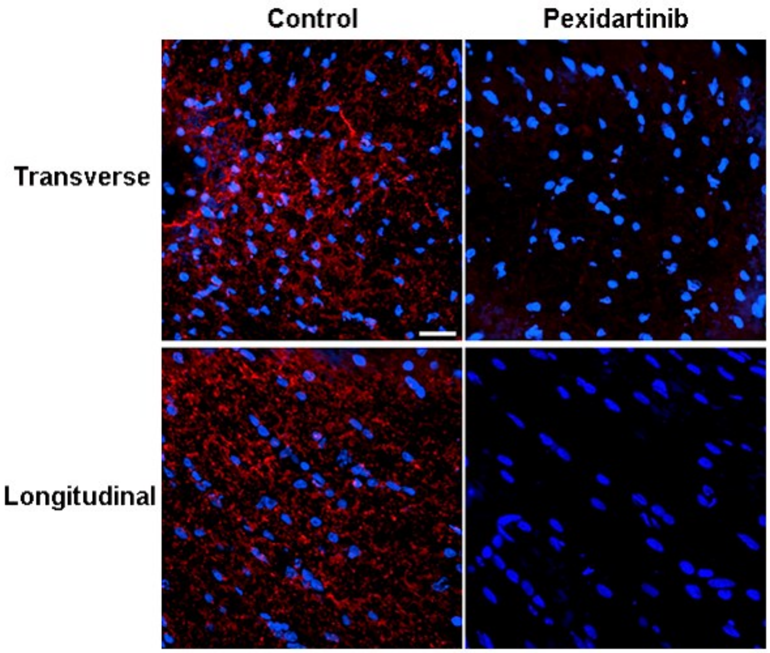

Supplementary Figure S1. Depletion of MNPs from the optic nerves of mice treated with pexidartinib. Representative images of transverse (top row) or longitudinal (bottom row) cryosections of optic nerves from P90 *Vglut2-Cre;ndufs4*$^{loxP/+}$ mice treated with control or pexidartinib-infused chow beginning at P25 and raised under normoxia. MNPs were immunolabeled for Iba1 (red) and nuclei labeled with DAPI (blue). Iba1 signal was robustly depleted from the nerves of pexidartinib-treated mice. Note that most of the Iba1 signal in the control sections is from MNP processes rather than cell bodies, the majority of which were not captured within the thin sections. Bar, 20 µm.
